# Model Predictive Regulation on Manifolds in Euclidean Space

**DOI:** 10.3390/s22145170

**Published:** 2022-07-10

**Authors:** Karmvir Singh Phogat, Dong Eui Chang

**Affiliations:** 1 Ernst & Young (EY) AI Lab, EY Office Garnet BTP, Bangalore 560048, India; karmvir.phogat@gds.ey.com; 2 School of Electrical Engineering, Korea Advanced Institute of Science and Technology, Daejeon 34141, Korea

**Keywords:** model predictive regulation, quadcopter tracking control, optimal exosystem tracking

## Abstract

One of the crucial problems in control theory is the tracking of exogenous signals by controlled systems. In general, such exogenous signals are generated by exosystems. These tracking problems are formulated as optimal regulation problems for designing optimal tracking control laws. For such a class of optimal regulation problems, we derive a reduced set of novel Francis–Byrnes–Isidori partial differential equations that achieve output regulation asymptotically and are computationally efficient. Moreover, the optimal regulation for systems on Euclidean space is generalized to systems on manifolds. In the proposed technique, the system dynamics on manifolds is stably embedded into Euclidean space, and an optimal feedback control law is designed by employing well studied, output regulation techniques in Euclidean space. The proposed technique is demonstrated with two representative examples: The quadcopter tracking control and the rigid body tracking control. It is concluded from the numerical studies that the proposed technique achieves output regulation asymptotically in contrast to classical approaches.

## 1. Introduction

One of the fundamental problems in control theory is to regulate the output of the plant, which is known in the literature as model predictive regulation (MPR) [1]. Consider a controlled nonlinear system on a manifold *M* as

$$\dot{x}=f\left(x,u,w\right)$$

with an exosystem

$$\dot{w}=a\left(w\right)$$

and the system output

y=h(x,u,w),

where x∈M,$u\in R^{m},$$w\in R^{d},$ and $y\in R^{p}.$ The optimal MPR problem is to determine a feedback control law u=κ(x,w) that regulates the system output asymptotically to zero, i.e.,

$$\lim_{t\to \infty }y\left(t\right)=0.$$

An optimal regulation control law is synthesized from an infinite horizon optimal control problem, i.e.,

$$\min_{u}\int_{0}^{\infty }l\left(x\left(t\right),u\left(t\right),w\left(t\right)\right)dt$$

subject to the controlled dynamics and a fixed initial condition $\left(x(0),w(0)\right)=\left(x_{0},w_{0}\right),$ where the control Lagrangian, $l:M\times R^{m}\times R^{d}\to R_{\geq 0}$, is sufficiently well-behaved and is zero if and only if y=0.

The output regulation problem for linear multivariate systems is formulated and its solvability conditions are derived, for the first time, by Francis in [2]. The regulation problem is further generalized for nonlinear systems [3] in which a feedforward control law is synthesized by solving the *Francis–Byrnes–Isidori* (FBI) partial differential equations (PDEs). The optimal output regulation problem is first studied by Krener [4], which proposes an optimal feedback control law synthesis in two steps: First, a feedforward control law is designed by solving the FBI PDEs using series solutions [5]. Second, using the feedforward control, the regulations problem is then translated to an optimal stabilization problem for designing an optimal feedback control law using the Al’brekht method [5]. In addition, Krener has developed a Nonlinear Systems Toolbox [6] for obtaining series solutions of the FBI PDEs and the *Hamilton–Jacobi–Bellman* (HJB) PDEs associated with the optimal stabilization problem [5].

Note that the FBI and the HJB PDEs are solved up to a polynomial approximation of a certain degree *d*, and therefore, the output regulation may not be achieved asymptotically if the output signal is not representable by a degree *d* polynomial. Due to such limitations of series solutions, output regulations may not be guaranteed for sophisticated output signals such as transcendental functions of the states. In this article, we address a class of output regulation problems in which the system states need to track signals generated by exosystems. For such output regulation problems, we derive novel FBI PDEs that are computationally efficient and achieve the output regulation asymptotically.

Another challenge is to extend the optimal output regulation problem to controlled systems on manifolds. A technique to deal with the output regulation problems on manifolds is to define equivalent output regulation problems on ambient Euclidean space and then employ well studied tools of optimal regulation on Euclidean space. A coherent way is to extend the system dynamics from the manifold to an ambient Euclidean space such that the manifold is a stable attractor of the extended system dynamics [7,8,9], and define the optimal regulation problem on that Euclidean space. An alternate approach is to define the regulation problem on local charts. This approach severely limits the applicability of the regulation problem due to local representation of the system dynamics. Moreover, the output regulation, under this approach, will only be limited to that particular local chart instead of the whole manifold.

In this article, we address two problems: First, we derive a reduced set of FBI PDEs for a class of output regulation problems on Euclidean space. We establish that, under the proposed approach, output regulation for a class of systems is achieved asymptotically by feedback control laws obtained via series solutions [5]. Second, we generalize the optimal regulation method on the Euclidean space to manifold via stable embedding of the controlled system dynamics on manifold into Euclidean spaces, and this optimal regulation problem was not addressed in [8]. Our proposed technique is then employed to the quadcopter tracking control and the rigid body attitude tracking system. It is observed from the numerical studies that the proposed approach provides better tracking performance as compared to the classical optimal regulation. Moreover, the controller built for the quadcopter in this paper is globally defined without any singularity, whereas the model predictive regulator designed in [10] is inherently locally defined since its use of Euler angles. This also demonstrates the superiority of our method. All the systems addressed in this article are continuous-time systems, in parallel to which there have been developments on model predictive regulation for discrete-time systems [1,11,12] and stochastic systems [13]. However, we do not treat either discrete-time or stochastic systems in this particle, leaving them as future research work.

This article unfolds as follows: Section 2 is devoted to a class of output regulation problems on Euclidean spaces and the derivation of the novel FBI PDEs. A generalized technique for the optimal output regulation of systems on manifolds is presented in Section 3. The proposed output regulation technique is well supported by the numerical studies conducted on representative examples of quadcopter tracking control and the rigid body attitude tracking system in Section 4. The concluding remarks and future directions are discussed in Section 5.

## 2. Optimal Output Regulation of Nonlinear Systems

Consider a nonlinear dynamic system

(1) $$\begin{array}{cc} \dot{x} & =f(x,u,w), \end{array}$$

(2) $$\begin{array}{cc} \dot{w} & =a(w), \end{array}$$

(3) $$\begin{array}{cc} y & =h(x,u,w), \end{array}$$

with the following data:

(a) plant state $x\in R^{n}$, plant input $u\in R^{m}$, exosystem state $w\in R^{d}$ and system output $y\in R^{p}$,
(b) $f:R^{n}\times R^{m}\times R^{d}\to R^{n}$ is a map depicting the plant dynamics (1) on $R^{n}$,
(c) $a:R^{d}\to R^{d}$ is a map depicting the exosystem dynamics (2) on $R^{d}$,
(d) $h:R^{n}\times R^{m}\times R^{d}\to R^{p}$ accounts for the system output (3).

An output regulation problem is to find a control law

(4) $$\begin{array}{cc} u: & \;R^{n}\times R^{d}\to R^{m}\\ \; & \left(x,w)\mapsto u(x,w\right) \end{array}$$

that steers, for any set of initial conditions $\left(x_{0},w_{0}\right)\in R^{n}\times R^{d}$, the system output of the nonlinear dynamics (1)–(3) asymptotically to zero, i.e.,

$$\lim_{t\to \infty }y\left(t\right)=0.$$

Output regulation problems are very commonly formulated for disturbance rejection and reference tracking by the system. The exosystem dynamics is, in general, designed to generate a reference signal or a modeled disturbance signal. Let us consider a case of reference tracking in which the plant output is the output of the plant dynamics (1) that does not include the exosystem $\tilde{h}\left(x,u\right)\in R^{p}$ for $\left(x,w\right)\in R^{n}\times R^{m}$, needs to track the reference signal, $q\left(w\right)\in R^{p}$ for $w\in R^{d}$, generated by the exosystem. Then, the system output

$$y=\tilde{h}\left(x,u\right)-q\left(w\right)$$

asymptotically converging to zero ensures that the plant output is asymptotically tracking the reference signal. In an identical manner, let us consider a case of disturbance rejection in which the plant output is stabilized asymptotically to zero while the plant dynamics (1) are subjected to the disturbance generated by the exosystem. Therefore, the system output

$$y=\tilde{h}\left(x,u\right)$$

is regulated to zero under the influence of the disturbance introduced in the plant dynamics.

Before we discuss necessary and sufficient conditions for the solvability of the output regulation problem, let us elucidate standard assumptions considered in the literature:

**Assumption** **1.** *The following assumptions for the nonlinear dynamics (1)–(3) hold:*

*(a)*  *The vector fields f and a and the map h are smooth.*
*(b)*  *For the control input u=0, the system dynamic (1)–(3) has an equilibrium point (x,w)=(0,0) such that the system output is zero, i.e., h(0,0,0)=0.*
*(c)*  *The equilibrium exosystem state w=0 of the exosystem (2) is stable and there exists a neighborhood $W_{0}$ containing zero, such that every initial condition $w\left(0\right)\in W_{0}$ is Poisson stable.*
*(d)*  *The linear approximation of the plant dynamics (1) is stabilizable at the equilibrium point (x,u,w)=(0,0,0), i.e., the pair*
$$A=\frac{\partial f}{\partial x}\left(0,0,0\right),\;\;B=\frac{\partial f}{\partial u}\left(0,0,0\right)$$
*is stabilizable.*

The output regulation problem with such a generality is difficult to solve in general. Therefore, the state feedback control law (4) is designed in an open neighborhood $O\subset R^{n}\times R^{d}$ of the origin at 0, such that for any initial condition $\left(x(0),w(0)\right)=\left(x_{0},w_{0}\right)\in O$, the system output (3) of the dynamics (1)–(3) converges at zero. The solvability condition for the output regulation problem is established by the following theorem:

**Theorem** **1** ([3]). *Under Assumption 1, there exist a neighborhood $O\subset R^{n}\times R^{d}$ of 0 and a $C^{k}\left(k\geq 2\right)$ state feedback, $u=u\left(x,w\right)\in R^{m}$ for (x,w)∈O, that asymptotically stabilizes the output of the system dynamics (1)–(3) to zero if and only if there exist $C^{k}$ mappings x=θ(w) with θ(0)=0, and u=λ(w) with λ(0)=0, both defined in a neighborhood $W\subset R^{d}$ of 0, such that*

(5) $$\begin{array}{cc} \; & \frac{\partial \theta }{\partial w}a\left(w\right)=f\left(\theta (w),\lambda (w),w\right), \end{array}$$

(6) $$\begin{array}{cc} \; & h\left(\theta (w),\lambda (w),w\right)=0. \end{array}$$

*for all w∈W.*

The PDE (5) with the algebraic constraints (6) is known in the literature as the FBI equation [3,4]. As a consequence of Theorem 1, for any initial condition $x\left(0\right)=\theta \left(w(0)\right)$ with w(0)∈W, the system dynamics (1)–(3) under the feedforward control u=λ(w) leads to

$$y\left(t\right)=h\left(\theta \left(w(t)\right),\lambda \left(w\left(t\right)\right),w\left(t\right)\right)=0\;\;\mathrm{for}\;t\geq 0.$$

Thus, the feedback control law for the output regulation can be designed as

(7) $$\begin{array}{c} u\left(x,w\right)=\lambda \left(w\right)+\kappa \left(x-\theta (w),w\right), \end{array}$$

where the feedback term κ with κ(0)=0 is derived to make the so-called output regulation manifold

$$M_{R}=\left\{\left.\left(x,w\right)\in R^{n}\times R^{d}\;\right|\;x-\theta \left(w\right)=0\right\}$$

a stable attractor.

The problem of synthesizing optimal feedback control laws for output regulation is first proposed by Krener [4,14], and that is generalized to model predictive regulations [15]. The feedback control law (7) using Krener’s method is designed in two steps:

(i) The feedforward control u=λ(w) and the output regulation manifold $M_{R}$ are designed by solving the FBI Equations (5) and (6).
(ii) For the feedback κ, the nonlinear dynamics (1)–(3) is defined in the new coordinates
  z=x−θ(w),v=u−λ(w)
  as
  (8) $$\begin{array}{cc} \; & \dot{z}=f\left(z+\theta (w),v+\lambda (w),w\right)-\frac{\partial \theta }{\partial w}a\left(w\right),\\ \; & \dot{w}=a\left(w\right). \end{array}$$
  Under these new coordinate changes, the output regulation problem (4) is posed as an optimal stabilization problem for asymptotic stabilization of the dynamics (8) to zero as
  (9) $$\begin{array}{cc} \min_{v} & \int_{0}^{\infty }l\left(z(t),v(t)\right)dt\\ \mathrm{subject}\;\mathrm{to} & \left\{\begin{array}{c} \mathrm{system}\;\mathrm{dynamics}\;(8),\\ \left(z(0),w(0)\right)=\left(z_{0},w_{0}\right), \end{array}\right. \end{array}$$
  where $\left(z_{0},w_{0}\right)$ is fixed and the smooth control Lagrangian
  (10) $$l:R^{n}\times R^{m}\to R_{\geq 0},\;\left(z,v\right)\mapsto l\left(z,v\right)$$
  satisfies l(z,v)=0 if and only if (z,v)=(0,0). Then, the feedback term κ in (7) is the feedback control law *v* obtained by solving the optimal control problem (9), i.e.,
  $$v=\kappa \left(z,w\right)=\kappa \left(x-\theta (w),w\right).$$

**Remark** **1.** *Note that the PDE (5) along with the algebraic constraints (6) is often solved approximately via finite series solutions [4,5]. Assume that the solution (θ,λ) of the PDE is approximated by polynomials of degree r of the form*

(11) $$\begin{array}{c} \theta^{\left(r\right)}\left(w\right)=\sum_{i=1}^{r}\theta^{\left[i\right]}\left(w\right),\\ \lambda^{\left(r\right)}\left(w\right)=\sum_{i=1}^{r}\lambda^{\left[i\right]}\left(w\right), \end{array}$$

*where $\gamma^{\left[i\right]}\left(\alpha \right)$ denotes a polynomial homogeneous of degree i in α. Then, under the change of coordinates*

$$z=x-\theta^{\left(r\right)}\left(w\right)\;\;\mathrm{and}\;\;v=u-\lambda^{\left(r\right)}\left(w\right),$$

*the optimal stabilization problem (9) leads to the following feedback control law*

(12) $$\begin{array}{c} {\bar{u}}^{\left(r\right)}\left(x,w\right)=\lambda^{\left(r\right)}\left(w\right)+\kappa \left(x-\theta^{\left(r\right)}\left(w\right),w\right) \end{array}$$

*that in turn ensures that the state-action pair (x,u) converges asymptotically to $\left(\theta^{\left(r\right)}\left(w\right),\lambda^{\left(r\right)}\left(w\right)\right)$. It is worth noting that $h\left(\theta^{\left(r\right)}\left(w\right),\lambda^{\left(r\right)}\left(w\right),w\right)$ may not be zero due to the approximation of the feedforward control u=λ(w) and the output regulation manifold $M_{R}$. Therefore, the series solution (12) does not guarantee asymptotic convergence of the system output (3) to zero. However, the output approximation error*

$$e_{y}\left(w\right)=h\left(\theta \left(w\right),\lambda \left(w\right),w\right)-h\left(\theta^{\left(r\right)}\left(w\right),\lambda^{\left(r\right)}\left(w\right),w\right)$$

*is of order $O{\left(w\right)}^{r+1}$ [4], Theorem 4.2.*

Equipped with a sufficient understanding of output regulation, let us design a feedback law for a class of nonlinear systems that leads to an asymptotic convergence of the system output to zero.

### 2.1. Problem Statement

Consider a nonlinear system

(13) $$\begin{array}{cc} {\dot{x}}_{1} & =f_{1}\left(x_{1},x_{2},u,w\right), \end{array}$$

(14) $$\begin{array}{cc} {\dot{x}}_{2} & =f_{2}\left(x_{1},x_{2},u,w\right), \end{array}$$

(15) $$\begin{array}{cc} \dot{w} & =a(w), \end{array}$$

(16) $$\begin{array}{cc} y & =x_{1}-\tilde{h}\left(w\right), \end{array}$$

where $x=\left(x_{1},x_{2}\right)\in R^{p}\times R^{n-p}$ is the plant state with vector field $f=(f_{1},f_{2})$ governing the plant dynamics, $w\in R^{d}$ is the exosystem state with vector field *a* governing the exosystem dynamics and $y\in R^{p}$ is the system output.

**Assumption** **2.** *Assumption 1 holds for the system dynamics (13)–(16) with the choice of $x=(x_{1},x_{2}),$$f=(f_{1},f_{2})$ and $h\left(x,u,w\right)=x_{1}-\tilde{h}\left(w\right)$ that brings the dynamics (13)–(16) to the standard form (1)–(3).*

Note that the system dynamics (13)–(16) is in standard form, and therefore, Theorem 1, leads to the following necessary and sufficient condition for the solvability of the output regulation problem for the system dynamics (13)–(16):

**Theorem** **2.** *Under Assumption 2, there exist a neighborhood $O\subset R^{n}\times R^{d}$ of 0 and a $C^{k}\left(k\geq 2\right)$ state feedback*

$$u:O\to R^{m},\;\left(x_{1},x_{2},w\right)\mapsto u\left(x_{1},x_{2},w\right)$$

*that asymptotically stabilizes the output of the system dynamics (13)–(16) to zero if and only if there exist $C^{k}$ mappings $x_{2}=\tilde{\theta }\left(w\right)$ with $\tilde{\theta }\left(0\right)=0$, and u=λ(w) with λ(0)=0, both defined in a neighborhood $W\subset R^{d}$ of 0, satisfying the conditions*

(17) $$\begin{array}{cc} \; & \frac{\partial \tilde{\theta }}{\partial w}a\left(w\right)=f_{2}(\tilde{h}\left(w\right),\tilde{\theta }\left(w\right),\lambda \left(w\right),w), \end{array}$$

(18) $$\begin{array}{cc} \; & \frac{\partial \tilde{h}}{\partial w}a\left(w\right)-f_{1}(\tilde{h}\left(w\right),\tilde{\theta }\left(w\right),\lambda \left(w\right),w)=0. \end{array}$$

**Proof.**  We know that the dynamics (13)–(16) with the choice of $x=\left(x_{1},x_{2}\right),f=\left(f_{1},f_{2}\right)$ and $h\left(x,u,w\right)=x_{1}-\tilde{h}\left(w\right)$ is in standard form (1)–(3). Hence, applying Theorem 1 to the dynamics (13)–(16) gives: There exists a neighborhood $O\subset R^{n}\times R^{d}$ of 0 and a $C^{k}\left(k\geq 2\right)$ state feedback

$$u:O\to R^{m},\;\left(x_{1},x_{2},w\right)\mapsto u\left(x_{1},x_{2},w\right)$$

that asymptotically stabilizes the output of the system dynamics (13)–(16) to zero if and only if there exist $C^{k}$ mappings $\left(x_{1},x_{2}\right)=(\theta_{1}\left(w\right),\tilde{\theta }\left(w\right))$ with $(\theta_{1}\left(0\right),\tilde{\theta }\left(0\right))=0$, and u=λ(w) with λ(0)=0, both defined in a neighborhood $W\subset R^{d}$ of 0, satisfying the conditions

(19) $$\begin{array}{cc} \; & \frac{\partial \theta_{1}}{\partial w}a\left(w\right)=f_{1}(\theta_{1}\left(w\right),\tilde{\theta }\left(w\right),\lambda \left(w\right),w), \end{array}$$

(20) $$\begin{array}{cc} \; & \frac{\partial \tilde{\theta }}{\partial w}a\left(w\right)=f_{2}(\theta_{1}\left(w\right),\tilde{\theta }\left(w\right),\lambda \left(w\right),w), \end{array}$$

(21) $$\begin{array}{cc} \; & \theta_{1}\left(w\right)-\tilde{h}\left(w\right)=0. \end{array}$$

The algebraic constraint (21) is satisfied if and only if $\theta_{1}\left(w\right)=\tilde{h}\left(w\right)$. Therefore, substituting $\theta_{1}=\tilde{h}$ in (19) leads to (18) and (20) leads to (17). This proves the assertion. □

**Remark** **2.** *Note that the PDE (17) and (18) with algebraic constraint is in the same form as (5) and (6); however, the dimension of the PDE (17) and (18) is reduced. Therefore, the reduced order PDE (17) and (18) is computationally efficient.*

We now turn to designing an optimal feedback control law using Krener’s method that locally regulates the system output (16) of the dynamics (13)–(16) asymptotically to zero.

First, a feedforward control law is designed by solving the FBI Equations (17) and (18) using HJB series solutions [4,5]. Let the series solution of the FBI Equations (17) and (18) be given by

(22) $$\begin{array}{c} x_{2}={\tilde{\theta }}^{\left(r\right)}\left(w\right),\;\;\mathrm{and}\;\;u=\lambda^{\left(r\right)}\left(w\right), \end{array}$$

where $\gamma^{\left(r\right)}\left(w\right)$ is a homogeneous polynomial in *w* up to degree *r*.

Second, the error dynamics is defined, under the change of coordinates

$$y=x_{1}-\tilde{h}\left(w\right),\;z=x_{2}-{\tilde{\theta }}^{\left(r\right)}\left(w\right),\;\;\mathrm{and}\;\;v=u-\lambda^{\left(r\right)}\left(w\right),$$

as

(23) $$\begin{array}{cc} \dot{y} & =f_{1}\left(y+\tilde{h}\left(w\right),z+{\tilde{\theta }}^{\left(r\right)}\left(w\right),\lambda^{\left(r\right)}\left(w\right),w\right)-\frac{\partial \tilde{h}}{\partial w}a\left(w\right),\\ \dot{z} & =f_{2}\left(y+\tilde{h}\left(w\right),z+{\tilde{\theta }}^{\left(r\right)}\left(w\right),\lambda^{\left(r\right)}\left(w\right),w\right)-\frac{\partial {\tilde{\theta }}^{\left(r\right)}}{\partial w}a\left(w\right),\\ \dot{w} & =a(w), \end{array}$$

and the output regulation problem is translated to a stabilization problem as

(24) $$\begin{array}{cc} \min_{v} & \int_{0}^{\infty }l\left(y(t),z(t),v(t)\right)dt\\ \mathrm{subject}\;\mathrm{to} & \left\{\begin{array}{c} \;\mathrm{system}\;\mathrm{dynamics}\;(23),\\ \left(y(0),z(0),w(0)\right)=\left(y_{0},z_{0},w_{0}\right), \end{array}\right. \end{array}$$

where $\left(y_{0},z_{0},w_{0}\right)$ is fixed and the smooth control Lagrangian

(25) $$l:R^{p}\times R^{n-p}\times R^{m}\to R_{\geq 0},\;\left(y,z,v\right)\mapsto l\left(y,z,v\right)$$

satisfies l(y,z,v)=0 if and only if (y,z,v)=0. The infinite horizon optimal control problem (24) is solved using Al’brekht’s method and the feedback control law

$$v=\kappa \left(y,z\right)=\kappa \left(x_{1}-\tilde{h}\left(w\right),x_{2}-{\tilde{\theta }}^{\left(r\right)}\left(w\right)\right)$$

is designed that locally stabilizes (y,z) asymptotically to zero [4], Theorem 4.2. Therefore, the optimal feedback control

(26) $$\begin{array}{c} {\tilde{u}}^{\left(r\right)}\left(x,w\right)=\lambda^{\left(r\right)}\left(w\right)+\kappa \left(x_{1}-\tilde{h}\left(w\right),x_{2}-{\tilde{\theta }}^{\left(r\right)}\left(w\right)\right) \end{array}$$

locally regulates the output of the dynamics (13)–(16) to zero asymptotically. The system output *y* converges asymptotically to zero due to the fact that the PDE series solutions (22) do not affect the output regulation manifold

$$M_{R}=\left\{\left(x_{1},x_{2},w\right)\in R^{n}\times R^{d}\left|\;x_{1}-\tilde{h}\left(w\right)=0\right.\right\}.$$

### 2.2. Computational Complexity

The feedback regulation problem for the system (13)–(16) is solved in two ways. A feedback control law is obtained by solving one of the FBI (5) and (6) and the FBI (17) and (18). As the dimension of the PDE in FBI (17) and (18) is reduced by *p* as compared to the FBI (5) and (6), it leads to a significant reduction in computation time. On the other hand, the regulation manifold of the system (13)–(16) is explicitly known and therefore, the feedback regulation law obtained by FBI (5) and (6) is more accurate as compared to FBI (17) and (18). A series solution of degree *r* of the FBI (5) requires the solving of a linear system of order $O\left(\left(n+m\right)d^{j}\right)$ recursively for each degree j=1,…,r. Therefore, the computation time for solving the FBI (17) and (18) using series solutions up to degree *r* is of order $O({\left(n+m\right)}^{3}d^{3r})$ and for the FBI (5) and (6) is of the order $O({\left(n-p+m\right)}^{3}d^{3r}).$ It can be concluded from the computation time analysis that there will be a significant reduction in computation time when the degree of the approximate series solution is large.

Let us now generalize the output regulation problem to manifolds. We know that many robotics and aerospace systems evolve on manifolds. The optimal stabilization theory developed by Krener [4] cannot be directly applied to the system evolving on manifolds. An intuitive way is to extended the system to the ambient Euclidean space and design the controller in that ambient space; however, such extensions may not preserve the stabilizability of the linearized system, which is one key assumption for the FBI Equations (5) and (6). This hurdle is circumvented by stably embedding the system dynamics into the ambient Euclidean space [8].

## 3. Output Regulation on Manifolds

Consider a class of nonlinear systems on a manifold $M\subset R^{n}$

(27) $$\begin{array}{cc} \dot{x} & =f(x,u,w), \end{array}$$

(28) $$\begin{array}{cc} \dot{w} & =a(w), \end{array}$$

(29) $$\begin{array}{cc} y & =h(x,u,w), \end{array}$$

where plant state x∈M, plant input $u\in R^{m}$, exosystem state $w\in R^{d}$ and system output y∈N such that the manifold $N\subset R^{n}$ is embedded in $R^{p}$ with p≤n.

The output regulation problem on the manifold is solved by stably embedding the system dynamics (27)–(29) to an appropriate Euclidean space such that the linearized system in the ambient Euclidean space is stabilizable. We would like to stress on the fact that the stabilizability of the linearized dynamics is one of the key assumptions for existence of an output regulating feedback control law; see Assumption 1.

A stabilizable extension of the dynamics (27)–(29) on the ambient Euclidean space $R^{n}$ is conducted in two steps [8]:

1. The plant dynamics (27) is extended to $R^{n}$ and the system output (29) is extended on $R^{p}$ as
  (30) $$\begin{array}{cc} \dot{x} & =f_{e}\left(x,u,w\right),\;x\in R^{n},\;u\in R^{m},\;w\in R^{d} \end{array}$$
  (31) $$\begin{array}{cc} \dot{w} & =a(w), \end{array}$$
  (32) $$\begin{array}{cc} y & =h_{e}\left(x,u,w\right),\;y\in R^{p} \end{array}$$
  such that $f_{e}\left(x,u\right)=f\left(x,u\right)$ and $h_{e}\left(x,u,w\right)=h\left(x,u,w\right)$ for all $\left(x,u,w\right)\in M\times R^{m}\times R^{d}$. As the extended plant dynamics (30) is identical to (27) on *M*, the manifold *M* is an invariant subset of $R^{n}$, i.e., for initial conditions $\left(x(0),w(0)\right)\in M\times R^{d}$, system trajectories of the dynamics (30)–(32) satisfy
  $$\left(x(t),w(t)\right)\in M\times R^{d}\;\;\mathrm{for}\;\mathrm{all}\;\;t.$$
2. Add a drift term to the extended plant dynamics (30) such that it is stabilizable in the transversal direction to *M* in $R^{n}.$ Suppose there exists a function $V:U\subset R^{n}\to R_{\geq 0}$ on open neighborhood *U* of *M* in $R^{n}$ such that
  $$M=V^{-1}\left(0\right),$$
  and
  $$\nabla V\left(x\right)\cdot f_{e}\left(x,u,w\right)=0\;\;\mathrm{for}\;\mathrm{all}\;\left(x,u,w\right)\in U\times R^{m}\times R^{d}.$$
  Therefore, the extended plant dynamics (30) is stably extended and that leads to the following linearly stabilizable extension of (27)–(29) on $U\times R^{m}\times R^{d}$:
  (33) $$\begin{array}{cc} \dot{x} & =\tilde{f}\left(x,u,w\right):=f_{e}\left(x,u,w\right)-\alpha \nabla V\left(x\right), \end{array}$$
  (34) $$\begin{array}{cc} \dot{w} & =a(w), \end{array}$$
  (35) $$\begin{array}{cc} y & =h_{e}\left(x,u,w\right), \end{array}$$
  where α>0. Here, instead of the number α>0, one can more generally use an n×n positive definite symmetric matrix-valued function. A detailed discussion on the transversal stability of *M* in the stably extended dynamics (33)–(35) may be found in [8].

The system dynamics (33)–(35) is defined in Euclidean space and therefore, Krener’s method for designing feedback control for the output regulation problem is directly applicable without any modification.

For the sake of clarity, let us consider an example of a single axis rotation of a rigid body. The state space of the dynamics is SO(2)×R where SO(2), (the set of 2×2 orthonormal matrices with determinant 1,) accounts for the attitude of the rigid body and the angular velocity of the body about the rotation axis lies in R. The manifold SO(2) is a Lie group and the set so(2), (the set of 2×2 real skew-symmetric matrices,) is its Lie algebra. The attitude dynamics for single axis rotation of the rigid body is given by

(36) $$\begin{array}{cc} \dot{R} & =R\hat{\Omega }, \end{array}$$

(37) $$\begin{array}{cc} J\dot{\Omega } & =\tau , \end{array}$$

where (R,Ω)∈SO(2)×R with *R* determines the attitude of the rigid body and Ω determines the angular velocity of the rigid body, $J\in R_{\geq 0}$ is the moment of inertia, τ∈R is the torque applied along the axis of rotation, and the hat map ∧:R→so(2) is the vector space isomorphism defined as follows: for β∈R

$$\hat{\beta }=\left[\begin{array}{cc} 0 & -\beta \\ \beta & 0 \end{array}\right].$$

Note that the manifold SO(2) is embedded in $R^{2\times 2}$ and therefore, the system dynamics (36) and (37) is naturally extended to the ambient space $R^{2\times 2}\times R$. However, such natural extensions may not guarantee the stabilization of its linearized dynamics around an equilibrium point of interest. Let us define a stable extension of the dynamics (36) and (37) in a neighborhood

$${\mathrm{GL}}^{+}\left(2\right)=\left\{X\in R^{2\times 2}\;\left|\;\det(X)>0\right.\right\}$$

of SO(2). To this end, let us define a Lyapunov-like function, $V:{\mathrm{GL}}^{+}\left(2\right)\times R\to R_{\geq 0}$ by

$$V\left(R,\Omega \right)=\frac{1}{4}{∥R^{⊤}R-I∥}^{2}$$

for $\left(R,\Omega \right)\in {\mathrm{GL}}^{+}\left(2\right)\times R$ with the usual Euclidean norm ∥·∥ on $R^{2\times 2}$, which satisfies

$$V^{-1}\left(0\right)=\mathrm{SO}\left(2\right),\;\mathrm{and}\;\nabla_{R}V\cdot \left(R\hat{\Omega }\right)=0.$$

It leads to a stable extension of the dynamics (36) and (37) on $R^{2\times 2}\times R$ as

(38) $$\begin{array}{cc} \dot{R} & =R\hat{\Omega }-\alpha R\left(R^{⊤}R-I\right), \end{array}$$

(39) $$\begin{array}{cc} J\dot{\Omega } & =\tau , \end{array}$$

where α>0. Let us consider an output regulation problem on the manifold SO(2), where the exosystem

$$\dot{w}=a\left(w\right)$$

generates attitude signals, $\tilde{h}\left(w\right)\in \mathrm{SO}\left(2\right)$ with $w\in R^{d}$, for the dynamics (36) and (37) to track. The system dynamics with an exosystem for the output regulation is defined as

(40) $$\begin{array}{cc} \dot{R} & =R\hat{\Omega }-\alpha R\left(R^{⊤}R-I\right), \end{array}$$

(41) $$\begin{array}{cc} J\dot{\Omega } & =\tau , \end{array}$$

(42) $$\begin{array}{cc} \dot{w} & =a(w), \end{array}$$

(43) $$\begin{array}{cc} y & =R-\tilde{h}\left(w\right). \end{array}$$

The dynamics (40)–(43) are defined in Euclidean space and therefore, the Krener’s method [4] for optimal regulation is readily applied to find a feedforward and feedback control law. Using Theorem 2, the feedforward control law, τ=λ(w) with $w\in R^{d}$, which makes the manifold $\Omega =\tilde{\theta }\left(w\right)$ invariant, needs to satisfy the following FBI equations

(44) $$\begin{array}{cc} \; & \frac{\partial \tilde{h}}{\partial w}a\left(w\right)=\tilde{h}\left(w\right)\hat{\tilde{\theta }\left(w\right)} \end{array}$$

(45) $$\begin{array}{cc} \; & J\frac{\partial \tilde{\theta }}{\partial w}a\left(w\right)=\lambda \left(w\right). \end{array}$$

**Remark** **3.** *Note that the FBI (44) and (45) is a PDE in R with algebraic constraints in $R^{2\times 2}$ in contrast to the FBI obtained using Theorem 1 that is a PDE in $R^{2\times 2}\times R$ with algebraic constraints in $R^{2\times 2}$. This simple example demonstrates that the PDE dimension is reduced to a large extent and it contributes to fast computation.*

**Remark** **4.** *Embedding SO(2) to $R^{2\times 2}$ increases the dimension of the state space by 3; however, one can identify SO(2) with the unit circle and embed the unit circle in $R^{2}$ (the ambient space) which only increases the dimension of the state space by 1.*

**Remark** **5.** *Note that the output regulation technique by Krener [4] does not incorporate state and control constraints. For output regulation of the safety critical systems where state and control constraints are crucial to consider at the controller design stage, a model predictive control approach is proposed by Krener [15]. The model predictive control approach is directly extended to manifolds by stably extending the system dynamics to an ambient Euclidean space.*

## 4. Simulation Results

Let us solve the output regulation problem for the bi-directional quadcopter [16] and the rigid body attitude motion with Krener’s Matlab-based Nonlinear Systems Toolbox [6]. We demonstrate with the quadcopter example that fairly complex problems can be handled using this approach.

### 4.1. Quadcopter

A bidirectional quadcopter is an unmanned aerial vehicle that is fitted with four rotors to generate bidirectional (upward and downward) thrust and a torque to orient the quadcopter. The system dynamics for the quadcopter is given by

(46) $$\begin{array}{cc} \dot{R} & =R\hat{\Omega }, \end{array}$$

(47) $$\begin{array}{cc} I\dot{\Omega } & =\hat{I\Omega }\Omega +\tau , \end{array}$$

(48) $$\begin{array}{cc} m\ddot{x} & =-mge_{3}+Re_{3}f \end{array}$$

where R∈SO(3) (the set of 3×3 rotation matrices) denotes the attitude, $\Omega \in R^{3}$ denotes the body angular velocity, and $x\in R^{3}$ defines the position of the quadcopter. The control inputs are *f* and τ, where f∈R accounts for the upward thrust generated by the rotors, and $\tau \in R^{3}$ is the torque applied on the body. The parameter *m* is the quadcopter mass, $e_{3}=\left(0,0,1\right),$ and I is the 3×3 moment of inertia matrix. The hat map $\wedge :R^{3}\to \mathrm{so}\left(3\right)$, where so(3) denotes the set of 3×3 skew symmetric matrices, is a vector space isomorphism defined as $\hat{x}y=x\times y$ for all $x,y\in R^{3}$.

Consider a position tracking problem in which the quadcopter traces a path

(49) $$\begin{array}{c} \tilde{h}\left(w\right)=\left(\begin{array}{c} w_{2}+2w_{1}w_{2}\\ w_{1}-2\left(w_{1}^{2}-w_{2}^{2}\right)\\ -3w_{2}+4w_{2}^{3} \end{array}\right)\in R^{3} \end{array}$$

that is generated by an exosystem

(50) $$\begin{array}{c} \dot{w}=aw, \end{array}$$

where

$$a=\left(\begin{array}{cc} 0 & -0.1\\ 0.1 & 0 \end{array}\right).$$

The tracking problem is to regulate the output

(51) $$\begin{array}{c} y=x-\tilde{h}\left(w\right) \end{array}$$

to zero that is subject to the quadcopter dynamics (46)–(48) and the exosystem dynamics (50). Note that the output regulation problem for the quadcopter dynamics (46)–(48) with the exosystem (50) and the output (51) is in the standard form. Therefore, Krener’s method extended to manifolds, as described in Section 3, is employed to design a feedback control law for regulating the output asymptotically to zero.

In order to apply Krener’s method, let us first stably extend the quadcopter dynamics (46)–(48) to Euclidean space in an identical manner as in (38) and (39). The stably extended dynamics with the exosystem and output is defined by

(52) $$\begin{array}{cc} \dot{R} & =R\hat{\Omega }-\alpha R\left(R^{⊤}R-I\right), \end{array}$$

(53) $$\begin{array}{cc} I\dot{\Omega } & =\hat{I\Omega }\Omega +\tau , \end{array}$$

(54) $$\begin{array}{cc} \dot{x} & =v, \end{array}$$

(55) $$\begin{array}{cc} \dot{v} & =-ge_{3}+\frac{f}{m}Re_{3}, \end{array}$$

(56) $$\begin{array}{cc} \dot{w} & =aw, \end{array}$$

(57) $$\begin{array}{cc} y & =x-\tilde{h}\left(w\right), \end{array}$$

where $R\in R^{3\times 3},\Omega \in R^{3},x\in R^{3},v\in R^{3},w\in R^{2},$ and $y\in R^{3}.$ The dynamics (52)–(57) is in standard form and therefore, a feedback control law (12) up to degree *r* is given by

(58) $$\begin{array}{c} {\bar{u}}^{\left(r\right)}=\left(\begin{array}{c} {\bar{\tau }}^{\left(r\right)}\\ {\bar{f}}^{\left(r\right)} \end{array}\right)=\lambda^{\left(r\right)}\left(w\right)+\kappa \left({\bar{z}}^{\left(r\right)},w\right) \end{array}$$

where the feedforward $\lambda^{\left(r\right)}$ and the stabilizing manifold ${\bar{z}}^{\left(r\right)}=\left(R,\Omega ,x,v\right)-\theta^{\left(r\right)}\left(w\right)$ are computed by solving the FBI (5) and (6) and the feedback κ is computed by solving the stabilization problem (9) using Al’brekht’s method. On the other hand, in our technique, the feedback control (26) up to degree *r* is given by

(59) $$\begin{array}{c} {\tilde{u}}^{\left(r\right)}=\left(\begin{array}{c} {\tilde{\tau }}^{\left(r\right)}\\ {\tilde{f}}^{\left(r\right)} \end{array}\right)={\tilde{\lambda }}^{\left(r\right)}\left(w\right)+\kappa \left(x-\tilde{h}\left(w\right),{\tilde{z}}^{\left(r\right)},w\right) \end{array}$$

where the feedforward ${\tilde{\lambda }}^{\left(r\right)}$ and the stabilizing manifold ${\tilde{z}}^{\left(r\right)}=\left(R,\Omega ,v\right)-{\tilde{\theta }}^{\left(r\right)}\left(w\right)$ are computed by solving the FBI (17) and (18) and the feedback κ is computed by solving the stabilization problem (24) using Al’brekht’s method. Let $\bar{x}\left\{r\right\}$ and $\tilde{x}\left\{r\right\}$ be the positions that are traced by the quadcopter (46)–(48) under the feedback ${\bar{u}}^{\left(r\right)}$ and ${\tilde{u}}^{\left(r\right)}$, respectively. Then, the corresponding tracking errors are given by

$$\bar{y}\left\{r\right\}=\bar{x}\left\{r\right\}-\tilde{h}\left(w\right)\;\;\mathrm{and}\;\;\tilde{y}\left\{r\right\}=\tilde{x}\left\{r\right\}-\tilde{h}\left(w\right).$$

The following parameters have been considered for the numerical experiments:

$$\begin{array}{cc} \; & m=0.468\mathrm{kg},\;g=9.81{\mathrm{ms}}^{2},\;I=23\times 10^{-4}\mathrm{diag}\left(1,1,2\right)\\ \; & R\left(0\right)=I_{3\times 3},\;\Omega \left(0\right)=\left(0,0,0\right),\;x\left(0\right)=\left(0.2,0.04,0.3\right)\\ \; & v(0)=(0,0,0),\;w(0)=(0.3,0.2). \end{array}$$

**Figure 1:**
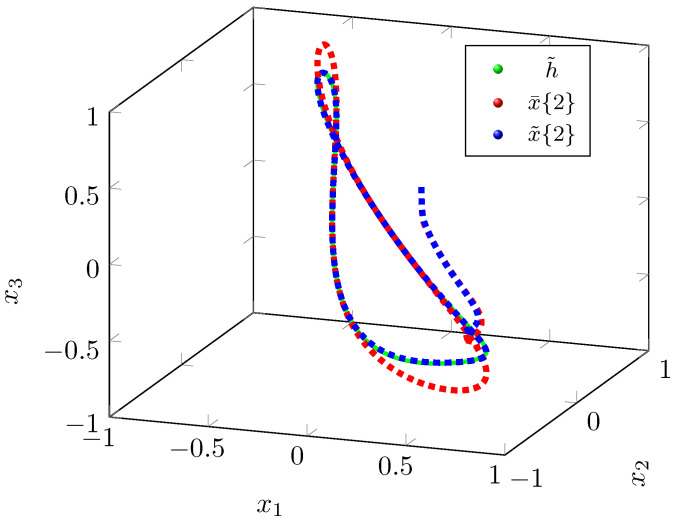
Phase portrait of quadcopter position.

**Figure 2:**
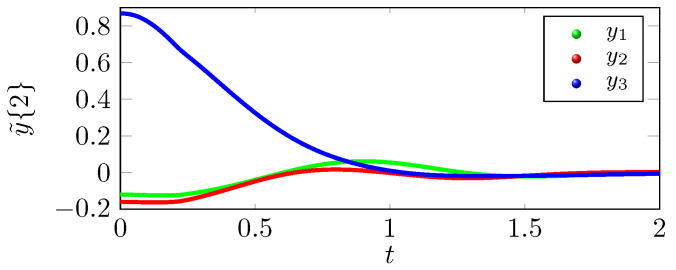
Tracking error with state feedback ${\tilde{u}}^{\left(2\right)}$.

**Figure 3:**
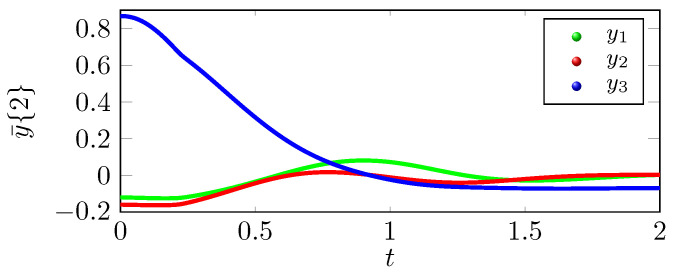
Tracking error with state feedback ${\bar{u}}^{\left(2\right)}$.

**Figure 4:**
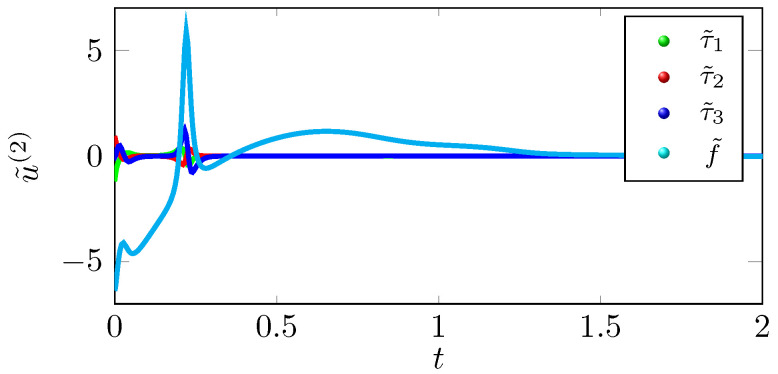
Optimal tracking control law ${\tilde{u}}^{\left(2\right)}$.

We can infer from the phase portrait in Figure 1 that the position trajectory $\tilde{x}\left\{r\right\}$ that is traced under the control law ${\tilde{u}}^{\left(r\right)}$ is tracing the exosystem trajectory $\tilde{h}$ more effectively as compared to the position trajectory $\bar{x}\left\{r\right\}$ that is traced under the control law ${\bar{u}}^{\left(r\right)}.$ The tracking errors $\tilde{y}\left\{r\right\}$ eventually converge to zero as shown in Figure 2, but $\bar{y}\left\{r\right\}$ does not converges to zero in Figure 3, which supports the claim that the tracking performance of the control law ${\tilde{u}}^{\left(2\right)}$ is better than ${\bar{u}}^{\left(2\right)}$. As shown in Figure 4, the optimal tracking control law ${\tilde{u}}^{\left(2\right)}$ shows that the quadcopter rotors will produce a negative thrust to catch up the exosystem trajectory and then torque and thrust eventually go to zero as the tracking error $\tilde{y}\left\{r\right\}$ goes to zero.

**Remark** **6.** *Note that the reference $\tilde{h}\left(w\right)$ generated by the exosystem (50) is a cubic polynomial in w, and therefore, the feedback controls ${\tilde{u}}^{\left(3\right)}$ and ${\bar{u}}^{\left(3\right)}$ are identical. However, the feedback control ${\tilde{u}}^{\left(2\right)}$ provides good performance and is computationally less intensive as compared to the feedback control ${\bar{u}}^{\left(3\right)}$.*

### 4.2. Rigid Body Attitude Control

A rigid body attitude dynamics is given by

$$\begin{array}{cc} \dot{R} & =R\hat{\Omega },\\ J\dot{\Omega } & =\hat{J\Omega }\Omega +\tau , \end{array}$$

where R∈SO(3) denotes the attitude, $\Omega \in R^{3}$ denotes the body angular velocity, the control input $\tau \in R^{3}$ is the torque applied on the body, and J is the 3×3 moment of inertia matrix. The hat map $\wedge :R^{3}\to \mathrm{so}\left(3\right)$ is the vector space isomorphism mentioned for the quadcopter example.

Consider a rigid body attitude tracking problem in which the rigid body is tracking an attitude profile,

(60) $$\begin{array}{c} \tilde{h}\left(w\right)=\exp\left({\hat{e}}_{s}w_{1}\right)\in R^{3} \end{array}$$

where $e_{s}={\left(1,1,1\right)}^{⊤}$ and $w=\left(w_{1},w_{2}\right)\in R^{2}$ that is generated by an exosystem on $R^{2}$ defined by

(61) $$\begin{array}{c} \dot{w}=aw \end{array}$$

with

$$a=\left(\begin{array}{cc} 0 & -1\\ 1 & 0 \end{array}\right).$$

**Figure 5:**
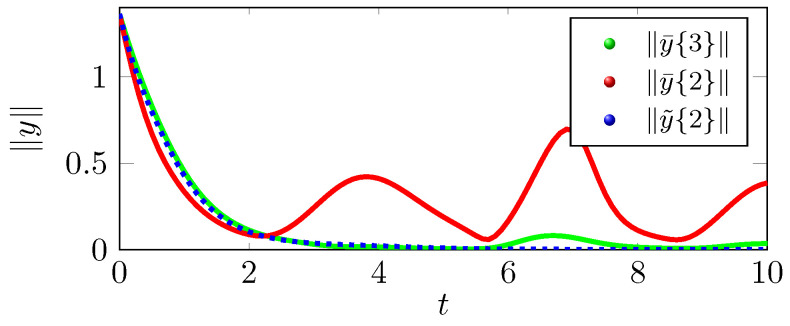
Tracking errors with three different controllers for the rigid body attitude control.

The output regulation problem for the rigid body tracking problem is to regulate the system output

$$\begin{array}{c} y=R-\tilde{h}\left(w\right) \end{array}$$

to zero asymptotically. We adopt an identical procedure, as in case of quadcopter control, to derive the feedback control ${\tilde{\tau }}^{\left(r\right)}$ and ${\bar{\tau }}^{\left(r\right)}$ as discussed in (58) and (59), respectively, and the corresponding tracking errors are given by $\tilde{y}\left\{r\right\}$ and $\bar{y}\left\{r\right\}$. Note that the rigid body tracking case is different from the quadcopter tracking in the sense that we are tracking a transcendental function (60). Therefore, the feedback control ${\bar{\tau }}^{\left(r\right)}$ cannot achieve regulation asymptotically for any *r*; however, the feedback control ${\tilde{\tau }}^{\left(r\right)}$ achieves regulation asymptotically for r=2; see Figure 5.

## 5. Conclusions and Future Works

This article presents a technique of designing an optimal feedback controller that achieves regulation asymptotically for a class of controlled systems. Moreover, we have generalized the optimal regulation problems on Euclidean spaces to manifolds with the embedding technique, and demonstrated its applicability by designing optimal tracking feedback control laws for the bi-directional quadcopter system and the rigid body control system. As a future work, we plan to investigate the case of model predictive regulation on Lie groups in the framework developed in this paper. We also plan to apply this framework to reinforcement learning.

## Data Availability

Not applicable.

## Abbreviations

The following abbreviations are used in this manuscript:

PDE	Partial differential equation
FBI	Francis–Byrnes–Isidori
MPR	Model predictive regulation
HJB	Hamilton–Jacobi–Bellman
